# Phylogenetically Distinct Bacteria Involve Extensive Dechlorination of Aroclor 1260 in Sediment-Free Cultures

**DOI:** 10.1371/journal.pone.0059178

**Published:** 2013-03-15

**Authors:** Shanquan Wang, Jianzhong He

**Affiliations:** Department of Civil and Environmental Engineering, National University of Singapore, Singapore, Singapore; Dowling College, United States of America

## Abstract

Microbial reductive dechlorination of the persistent polychlorinated biphenyls (PCBs) is attracting much attention in cleanup of the contaminated environment. Nevertheless, most PCB dechlorinating cultures require presence of sediment or sediment substitutes to maintain their dechlorination activities which hinders subsequent bacterial enrichment and isolation processes. The information on enriching sediment-free PCB dechlorinating cultures is still limited. In this study, 18 microcosms established with soils and sediments were screened for their dechlorination activities on a PCB mixture – Aroclor 1260. After one year of incubation, 10 out of 18 microcosms showed significant PCB dechlorination with distinct dechlorination patterns (e.g., Process H, N and T classified based on profiles of PCB congeners loss and new congeners formation). Through serial transfers in defined medium, six sediment-free PCB dechlorinating cultures (i.e., CW-4, CG-1, CG-3, CG-4, CG-5 and SG-1) were obtained without amending any sediment or sediment-substitutes. PCB dechlorination Process H was the most frequently observed dechlorination pattern, which was found in four sediment-free cultures (CW-4, CG-3, CG-4 and SG-1). Sediment-free culture CG-5 showed the most extensive PCB dechlorination among the six cultures, which was mediated by Process N, resulting in the accumulation of penta- (e.g., 236-24-CB) and tetra-chlorobiphenyls (tetra-CBs) (e.g., 24-24-CB, 24-25-CB, 24-26-CB and 25-26-CB) via dechlorinating 30.44% hepta-CBs and 59.12% hexa-CBs after three months of incubation. For culture CG-1, dechlorinators mainly attacked double flanked *meta*-chlorines and partially *ortho*-chlorines, which might represent a novel dechlorination pattern. Phylogenetic analysis showed distinct affiliation of PCB dechlorinators in the microcosms, including *Dehalogenimonas* and *Dehalococcoides* species. This study broadens our knowledge in microbial reductive dechlorination of PCBs, and provides essential information for culturing and stimulating PCB dechlorinators for *in situ* bioremediation applications.

## Introduction

The use of harmful PCB mixtures (e.g., Aroclor 1260) in electrical transformers, hydraulic fluids and other areas has resulted in PCB contamination in soils and sediments of lakes, rivers, and harbors worldwide [1]–[4]. Due to their low vapor pressure and water solubility, the refractory PCBs may enter the food-chains through bioaccumulation [5], thus posing a threat to the health of human beings and ecosystems [6]. Removal of halogenated compounds from the contaminated sites can be achieved using various remediation technologies including natural attenuation, physical methods (e.g. capping), thermal and chemical treatment (e.g. microwave-generated steam technology and photocatalytic dechlorination), and biological processes employing bacteria or yeast [5], [7]. Among them, bioremediation is an effective and economical approach to remove PCBs from contaminated environments, and the microbial removal of highly chlorinated PCB congeners is conducted anaerobically through microbial reductive dechlorination [8].

After the first report of *in situ* dechlorination of PCBs by anaerobes [9], [10], evidence to date have demonstrated that *in situ* PCB dechlorination is widespread in many anaerobic PCB contaminated environments, including freshwater (pond, lake, and river), estuarine, and marine sediments [11]. To understand the potential for bioremediation at these sites, it is important to evaluate the possible indigenous dechlorinators, which is normally conducted by culturing microbes in the laboratory through establishing microcosms with indigenous soil or groundwater samples [12]. Thus far, many enrichment cultures have been obtained by using either single PCB congeners or PCB mixtures [11], [13]. For example, a mixed culture enriched from Dutch sediment can dechlorinate 234-234-CB and 236-236-CB to penta- and tetra-CBs by attacking *ortho*, *meta*, or *para*-chlorines [14]. Two anaerobic PCB-dechlorinating enrichments with *para*-dechlorination specificities were obtained with sediments derived from Aroclor 1260-contaminated Woods Pond and PCB-free Sandy Creek Nature Center sediments, respectively [15]. Enriched cultures with extensive Aroclor 1260 dechlorination activity were also obtained by using effective primer compounds, e.g., 23456-CB [16] and brominated biphenyls [17]. Based on profiles of PCB congeners loss and new congeners formation by these enriched cultures and *in situ* field studies, eight distinct microbial dechlorination processes (i.e., Processes M, Q, H′, H, P, N, LP and T) have been identified [11], [18].

However, most of previously developed enrichment cultures require the presence of sediments to maintain PCB dechlorination activities [8], [11]. To further elucidate PCB dechlorination processes, isolation of PCB dechlorinators is necessary, which requires the development of sediment-free cultures [8]. To date, only a few sediment-free cultures exhibiting PCB dechlorination activity have been reported, e.g., two cultures were enriched on 2356-CB and 2345-CB by removing *ortho*-chlorines [19] and *meta*-/*para*-chlorines [20], respectively. Their PCB dechlorinators were subsequently identified to be *Chloroflexi* bacterium *o*-17 [21] and DF-1 [22] by using the PCR-DGGE method. Another tetrachloroethylene (PCE) - dechlorinating *Dehalococcoides mccartyi* strain 195 can also dechlorinate 23456-CB to 2346-, 2356- and 246-CBs [23]. However, 2356-CB, 2345-CB and 23456-CB are PCB congeners chlorinated on a single ring, which are not the major PCBs present at the contaminated sites [24]. For microbial reductive dechlorination of PCB mixtures (e.g. Aroclor 1260), it is more complicated to develop sediment-free cultures. For example, pure culture bacterium DF-1 isolated from 2345-CB dechlorination was also shown to extensively dechlorinate weathered PCB mixtures, nevertheless, in the presence of soil [25], [26]. Until now, only culture JN has been successfully established for PCB mixture dechlorination in sediment-free form [27], in which *Dehalococcoides* species was identified to couple their growth with Aroclor 1260 dechlorination by Process N [28]. This study made a major progress to show that PCB dechlorinating bacteria can maintain their Aroclor 1260 dechlorination activities in defined mineral medium. In culture JN, the activities required the presence of silica powder as a carrier for Aroclor 1260 to increase the bioavailability [27]. By using the same strategy, *Dehalococcoides* sp. CBDB1, a pure culture dechlorinating chlorobenzenes, also showed the capability to dechlorinate Aroclor 1260 in a co-metabolic process after pre-growing on trichlorobenzenes [29]. The requirement of silica powder as a sediment-substitute may explain one key role of sediments in PCB dechlorinating cultures is to improve the bioavailability of PCB mixtures. However, employing silica sediment-substitute shall complicate the medium preparation and operation processes for enriching and isolating PCB dechlorinators. Thus far, all the PCB dechlorinating bacteria were identified to be either *Dehalococcoides* species (i.e. Pinellas subgroup) or phylogenetically related but distinct *Chloroflexi* bacteria (e.g., DF-1 and *o*-17) [8], [30]. Therefore, information still remains limited on both maintaining Aroclor 1260 dechlorination activities in sediment-free cultures without the aid of sediment-substitutes and identification of their PCB dechlorinators.

In this study, 18 soil and sediment samples were collected from different locations to set up microcosms for screening of Aroclor 1260 dechlorinating microbes. From them, six sediment-free cultures with distinct dechlorination specificities were successfully established without amending any sediments/sediment-substitutes. Initial phylogenetic insights into key dechlorinators were also gained by using 16S rRNA gene-based techniques. In the sediment-free cultures, the growth of PCB dechlorinators coupled with Aroclor 1260 dechlorination was quantified by using real-time PCR.

## Materials and Methods

### Ethics Statement

No specific permits were required for the described field studies. The sampling activities did not involve any endangered or protected species, and sampling locations are not privately-owned or protected in any way.

### Chemicals

Unless stated otherwise, chemicals were purchased from Sigma-Aldrich at the highest purity available. All PCBs were purchased from AccuStandard (New Haven, CT, USA). H_2_ was obtained from a hydrogen generator (NM-H250, Schmidlin-DBS AG, Neuheim, Switzerland). The DNA extraction kits were obtained from Qiagen (QIAGEN, Hilden, Germany), and the GoldTaq DNA polymerase and related PCR reagents were purchased from Applied Biosystems (Foster City, CA, USA).

### Microcosm preparation, culture transferring, and growth conditions

A total of 18 samples were collected in four Asian countries, China (Wuhan in Hubei and Guiyu in Guangdong, China), Indonesia (West Java, Indonesia), Malaysia (Penang, Malaysia), and Singapore (Jurong Island, Singapore). The characteristics of samples are shown in Table 1. The sublayer soil and sediment samples (sampling depth, 5–20 cm) were acquired directly by filling sterile 50-ml plastic Falcon tubes that were capped and transported to the laboratory at an ambient temperature. Concentrations of PCBs in these samples were below detection limit. To control exposure of the samples to air, Falcon tubes were sealed with Parafilm (Pechiney Plastic Packaging Company, Chicago, IL, USA), and microcosm setup was conducted in anaerobic chamber as described soon after their arrivals [31]. Briefly, 90 ml of bicarbonate-buffered mineral salts medium amended with 10 mM of lactate were dispensed into 160 ml serum bottles containing ∼10 grams of collected samples [32], [33]. The mineral salts medium contains salts, trace elements and vitamins as shown in Table S1. L-cysteine and Na_2_S·9H_2_O (0.2 mM each) were added to the medium to achieved reduced conditions. The bottles were sealed with black butyl rubber septa (Geo-Microbial Technologies, Inc, Ochelata, OK, USA) and secured with aluminum crimp caps. After that, a 60 µl of Aroclor 1260 stock solution (50 mg of total PCBs per ml) in GC grade isooctane was spiked into the medium to a final nominal concentration of 30 ppm (or 81 µM). The microcosms were incubated in the dark at 30°C. PCB dechlorination activities were measured every four weeks by gas chromatograph equipped with an electron capture detector (GC-ECD) as described in the following section. Cultures were transferred when observing obvious PCB dechlorination activity. After three years, a total of six sediment-free cultures were obtained after at least 5 times of transferring supernatant of the active microcosm to the same medium (5%, v/v) as described above. Cultures amended with two individual PCB congeners (i.e., 2345-245-CB and 234-245-CB) were also prepared to determine their dechlorination pathways in the sediment-free cultures. All experiments were set up in triplicates. Duplicate abiotic controls and non-PCB controls were also set up for each experiment under the same conditions but without bacterial inocula and PCBs injection, respectively.

**Table 1:** Dechlorination of Aroclor 1260 in microcosms after 12 months of incubation.

Microcosm	Sample and its collection site	Mol% Change of PCB homolog^a^							Decreased PCB congener(s)^b^	Increased PCB congener(s)^c^
		Nona-CB	Octa-CB	Hepta-CB	Hexa-CB	Penta-CB	Tetra-CB	Tri-CB		
CW-1	Clay and silt around a fishing plant, Yang Tze River	0.00	−0.49	−8.94	−11.14	14.06	6.47	0.04	245-245-CB, 234-245-CB, 2345-245-CB, 2345-236-CB, 2345-25-CB	235-25-CB, 25-25-CB, 245-24-CB, 235-245-CB, 24-25-CB
CW-2	Sand and silt at Yang Tze River bank	0.00	−0.48	−8.71	0.39	8.00	0.80	0.00	2345-245-CB, 234-245-CB, 2345-25-CB	235-245-CB, 245-24-CB, 235-25-CB
CW-3	Digester sludge of a wastewater treatment plant of a pesticide factory	−0.06	−1.99	−15.00	−0.37	16.32	1.10	0.05	2345-245-CB, 234-245-CB, 2345-236-CB, 2345-234-CB, 2345-25-CB, 2346-245-CB	235-245-CB, 245-24-CB, 235-25-CB, 2345-26-CB, 245-25-CB
CW-4	Silt around wastewater treatment plant outlet of a pesticide factory	0.00	−1.08	−13.74	−15.1	15.39	14.05	0.48	245-245-CB, 234-245-CB, 2345-245-CB, 2345-236-CB, 2345-25-CB, 2345-234-CB	25-25-CB, 235-25-CB, 24-25-CB, 2345-26-CB
CG-1	Sand and silt near Liangjiang River	−0.01	−1.92	−14.96	2.43	13.75	0.71	0.00	2345-245-CB, 234-245-CB, 2345-236-CB, 234-236-CB, 2345-25-CB, 2345-234-CB	245-245-CB, 245-24-CB, 235-34-CB, 236-245-CB, 236-24-CB, 2356-245-CB
CG-2	Silt and clay at Liangjiang River bank	0.00	−0.20	−9.89	−1.92	10.79	1.20	0.02	2345-245-CB, 234-245-CB, 2345-25-CB	235-245-CB, 245-24-CB, 235-25-CB
CG-3	Sand of ditch sediment near electronic waste dump site	0.00	−0.72	−11.44	−2.09	11.45	2.80	0.00	2345-245-CB, 234-245-CB, 2345-236-CB, 2345-25-CB	245-24-CB, 235-245-CB, 236-245-CB
CG-4	Sand and silt of ditch sediment near electronic waste dump site	0.00	−0.03	−10.27	−1.56	11.39	0.47	0.00	2345-245-CB, 234-245-CB, 2345-25-CB, 2345-236-CB, 2345-234-CB	234-245-CB, 245-24-CB, 2345-26-CB, 235-25-CB
CG-5	Sand of ditch sediment near electronic waste dump site	−0.01	−0.01	−11.45	−28.95	14.94	21.02	4.46	245-245-CB, 236-245-CB, 234-245-CB, 2345-245-CB, 234-236-CB, 2345-245-CB, 234-236-CB, 2345-25-CB, 2345-236-CB, 236-25-CB	24-24-CB, 2356-24-CB, 236-24-CB, 24-24-CB, 24-25-CB, 24-26-CB, 24-2-CB
ID-1	Sand of Cipanas River Sediment	0.00	−0.03	−1.62	−0.56	1.84	0.37	0.00	ND	ND
MY-1	Soil of an orchard	0.00	−0.01	−1.10	−0.23	1.21	0.13	0.00	ND	ND
SG-1	Digester sludge of an industrial wastewater treatment plant	0.00	−1.28	−12.18	−10.14	14.94	8.61	0.05	245-245-CB, 2345-245-CB, 234-245-CB, 2345-236-CB, 2345-234-CB, 2345-25-CB	235-245-CB, 235-25-CB, 245-25-CB, 24-25-CB, 25-25-CB, 2345-26-CB

a “-“, decrease of mol%, all the mol% change of PCB homologs were measured quantitatively based on decrease or increase of congener peaks compared to control samples.

b PCB congeners were listed from more to less decrease, but all are above 2 mol% decrease.

c PCB congeners were listed from more to less increase, but all are above 2 mol% increase.

ND, no PCB congeners experienced more than 2 mol% decrease or increase.

### Analytical methods

PCBs in the culture medium were extracted with the solvent isooctane. Mixed liquor of 1 ml was withdrawn and subjected to a liquid-liquid extraction with an equal volume of isooctane in a 4 ml amber vial. The vial was vigorously shaken for 2 h and then centrifuged at 14,000 rpm for 10 min. The solvent phase of 0.5 ml was transferred to a 2-ml amber glass vial for subsequent GC analysis. PCBs were measured with a gas chromatograph (GC) 6890 N equipped with an electron capture detector (GC-ECD) (Agilent, Santa Clara, CA, USA) and a DB-5 capillary column (30 m×0.32 mm×0.25 µm film thickness; J&W Scientific, Folsom, CA, USA). The temperature program was initially held at 170°C for 5 min, increased at 2.5°C min^−1^ to 260°C, and held for 10 min. Injector and detector temperature were set at 250°C and 300°C, respectively. Nitrogen was used as a carrier gas at a flow rate of 1.2 ml min^−1^. Sample of one µl was injected into the GC inlet in a splitless mode. The elution time of all 209 PCB congeners was determined with PCB congener mixtures 1 through 9 from AccuStandard. The relative elution time of the PCBs in these mixes were published for DB-5 column [34]. PCBs were quantified by using a customized calibration standard prepared from Aroclor 1260 plus the congeners that are known to be frequent dechlorination products as described [35]. Additional congeners were quantified from standards prepared from the AccuStandard PCB congener mixtures. In all cases we used a seven-point calibration curve. The total moles of PCBs kept roughly the same throughout the whole dechlorination process. The microbial reductive dechlorination of PCBs result in profile changes of mol% of PCB congeners, which can be quantified based on their weight percent distributions and molecular weight as described previously [13], [27], [36]. Mole percent value for each congener, total number of chlorines per biphenyl and the PCB homolog distribution were calculated as described previously [17].

### DNA extraction, PCR, and sequencing

Total community DNA was extracted from 1 ml of cell pellets collected from dechlorinating cultures as well as the controls according to the manufacturer' instructions but with minor modifications [37]. The concentration of the nucleic acid was determined by a Nanodrop-1000 instrument (NanoDrop Technologies, Wilmington, DE, USA). PCR amplifications of 16 S rRNA gene sequences were conducted on a Mastercycler®cycler (Eppendorf, Hamburg, Germany) under conditions as described previously [33]. The primers used in this study are shown in Table S2. PCR products were sequenced and aligned as described previously [38].

### Two step DGGE (2S-DGGE)

2S-DGGE was developed to obtain full-length 16 S rRNA gene sequences of minor populations which might be difficult to be recovered from clone libraries [39]. PCR amplifications were first conducted with 8F and genus-specific reverse primer (i.e., DEB630R, DHC710R and DHCG812R) on community DNA of each culture. Then the diluted PCR products (× 50 dilutions) of *Dehalobacter/Dehalococcoides/Dehalogenimonas* 16 S rRNA gene sequences were subjected to subsequent 2S-DGGE procedures [39]. PCR products (12 µl) amplified with the GC-clamped universal primer sets were separated on an 8% polyacrylamide gel with a gradient range of 30–60% (100% denaturant consisted of 7 M urea and 40% deionized formamide) in 0.5 × TAE buffer. Gradient gels were cast with Bio-Rad's Model 475 gradient delivery system (Bio-Rad, Hercules, CA, USA). The electrophoresis was performed for 15 h at a constant electric current of 30 mA and a temperature of 60°C with the D-Code Mutation Detection System (Bio-Rad, Hercules, CA, USA). Gel images of DNA stained with SYBR® Gold (Invitrogen, Carlsbad, CA, USA) were taken by using a Molecular Imager Gel Doc XR System (Bio-Rad, Hercules, CA, USA). The DNA bands were excised and their DNA fragments were extracted by using the QIAEX II Gel Extraction Kit (QIAGEN, GmbH, Germany). The captured DNAs were then PCR re-amplified, and re-analyzed by DGGE to confirm that single bands were obtained before sending the PCR re-amplified products for sequencing. Based on sequencing data (from base 8–529, *E. coli* numbering), strain-specific forward primers would be designed or chosen from currently available primers for PCR amplification of the left 16 S rRNA gene sequence (from base 529–1392, *E. coli* numbering). Second round DGGE was employed to confirm the PCR amplification specificity. Lastly, the second round specifically amplified PCR products were sent for sequencing, and the full-length 16 S rRNA gene sequences can be obtained by assembling the two partial sequences.

### Illumina high throughput sequencing analysis of 16 S rRNA genes

To analyze the taxonomic composition of the sediment-free PCB dechlorinating cultures, the V3 region of the 16 S rRNA gene (from base 334–533, *E.coli* numbering) was chosen for PCR amplification with the eubacteria primer sets containing barcode sequences, 341F (5′-Fusion A-Barcode-ACTCCTACGGGAGGCAGCAG-3′) and 533R (5′-TTACCGCGGCTGCTGGCAC-3′). A total of six bar-coded [40] forward primers were used to differentiate the individual samples. Amplified PCR products were purified by using QIAquick PCR purification kit (QIAGEN, GmbH, Germany) according to the manufacturer's instructions. Then the six PCR samples with equal amounts were mixed together for subsequent Illumina high throughput sequencing. Illumina (Highseq2000, Illumina, San Diego, CA, USA) high throughput sequencing services were provided by BGI (Hongkong, China). Raw sequencing reads were checked for their quality through elimination of sequences that did not perfectly match the proximal PCR primer and that with short sequencing length (<130 nt). A total of 287,069 pair-end reads were obtained for these PCB dechlorinating cultures, and each sequence has an average read length of 150 bp. Pair-end reads were combined to form longer composite reads (170∼200 bp) by using the SHERA [41] software package. Sequence alignments were conducted with each subset reads based on NAST [42], and with other settings kept at their default values as described [43]. After NAST alignment, aligned subsets were merged into one Microsoft Excel file, in which sequences were clustered (based on 97% of sequence similarity) according to template ID. Manual adjustments were performed to improve the alignment and clustering whenever necessary. Representative sequences for each cluster were identified through Classifier [44] and BLAST analysis [45], which were further utilized to construct phylogenetic tree by using MEGA4 [46]. Relative abundances of predominant bacterial genera were showed by using BAND [47].

### qPCR

A TaqMan®quantitative real-time PCR (qPCR) (ABI 7500 Fast real-time PCR system; ABI, Foster City, CA, USA) assay was performed in triplicates for PCB dechlorinating cultures by using *Bacteria* and *Dehalococcoides* 16 S rRNA gene-targeted primers/probes, respectively [37], [38]. *Dehalogenimonas* species was quantified by using SYBR® green assays and *Dehalogenimonas* 16 S rRNA gene-targeted primers [48]. The primer and probe sequences used in this study were shown in Table S2. A calibration curve was obtained by using 10-fold serial dilutions of known plasmid DNA concentrations. The standard curves spanned a range of 10^2^ to 10^8^ gene copies per µl of template DNA. Nuclease-free water or plasmid without an insert was used as the negative control. The cell growth supported by chlorine removal was calculated using cell-growth (cells/ml)/total chlorine removal (nmol/ml).

### Sample nomenclature

In this study, the samples were denoted based on the locations from which they were collected. The abbreviation used for locations were as follows: CW, Wuhan, China; CG, Guiyu, China; ID, Indonesia; MY, Malaysia; SG, Singapore. For example, CW-4 indicates a sample from Wuhan, China.

### Nucleotide sequence accession numbers

The nucleotide sequence data obtained in this study were submitted to the Genbank with the following accession numbers: JQ990318-JQ990328.

## Results

### Screening PCB dechlorinating cultures

Soil and sediments collected from 4 locations were used as inocula in microcosm studies to screen their capabilities of dechlorinating PCB congeners in the commercial PCB mixture, Aroclor 1260. After one year of incubation, 12 out of 18 microcosms showed PCB dechlorination activities (Table 1), which predominantly dechlorinated hepta-CBs (e.g., 2345-245-CB, 2345-236-CB, 2345-234-CB and 2346-245-CB) and hexa-CBs (e.g., 234-245-CB, 2345-25-CB, 245-245-CB, 234-236-CB and 236-245-CB) to penta-CBs (e.g., 235-25-CB, 245-24-CB, 245-25-CB and 236-24-CB) and tetra-CBs (e.g., 24-25-CB, 25-25-CB and 24-24-CB). Several hexa-CB congeners were produced as intermediates, e.g., 235-245-CB, 234-245-CB and 236-245-CB. 10 out of the 12 active microcosms showed significant PCB dechlorination activities with distinct PCB dechlorination patterns. For example, 25-25-CB and 24-25-CB were the major tetra-CBs produced in CW-1, CW-4 and SG-1 microcosms, which are the major dechlorination products via PCB dechlorination Process H [11], [18]. In comparison, 24-24-CB and 24-25-CB were dominant tetra-CBs observed in CG-5 microcosm, which are usually generated via PCB dechlorination Process N [11], [18]. Interestingly, as shown in Table 1, six microcosms (i.e., CW-2, CW-3, CG-1, CG-2, CG-3 and CG-4) which preferred removing chlorines from 2345-245-CB did not dechlorinate 245-245-CB effectively, and almost all dechlorination activities stopped at penta-CBs (e.g., 245-24-CB and 235-25-CB). Other four highly active microcosms (i.e., CW-1, CW-4, CG-5 and SG-1) can effectively dechlorinate both 245-245-CB and 2345-245-CB mainly to penta- and tetra-CBs. Among the 12 active microcosms, culture CG-5 showed the most extensive PCB dechlorination capability.

### Development of sediment-free PCB dechlorinating cultures

To obtain sediment-free PCB dechlorinating cultures, the 12 active microcosms were transferred in defined medium amended with Aroclor 1260 (30 ppm or 81 µM) and lactate (10 mM). Subsequently, six sediment-free PCB dechlorinating cultures (i.e., CW-4, CG-1, CG-3, CG-4, CG-5 and SG-1) were obtained after five serial transfers (5% inocula, v/v). The PCB dechlorination activities can be sustained and transferred in defined medium amended with lactate. However, upon replacing lactate with acetate (10 mM) and H_2_ (5×10^4^ Pa or 0.40 mM), no PCB dechlorination activities were observed in the 6 sediment-free cultures after two transfers. For the six sediment-free cultures, two PCB congeners (i.e., 2345-245-CB and 234-245-CB, two of the most abundant congeners in Aroclor 1260 and comprising ∼20% by mole of total PCBs) were then spiked to the cultures to study their PCB dechlorination pathways. The data of mole percent value for each congener and PCB homolog distribution were collected after 6 months of incubation for cultures CW-4, CG-1, CG-3, CG-4 and SG-1; and after 3 months for culture CG-5.

**Figure 1:**
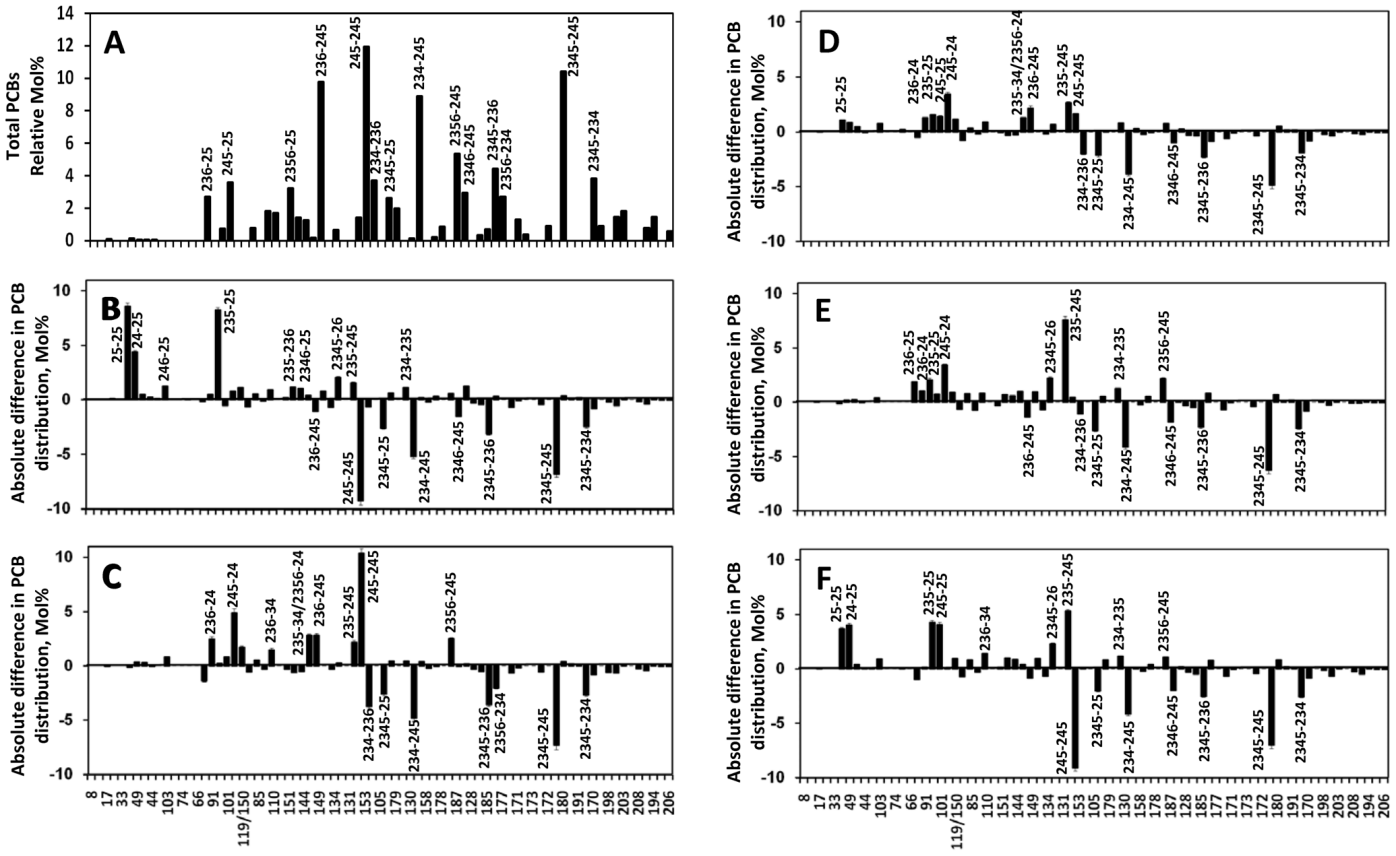
Reductive dechlorination of Aroclor 1260 in sediment-free cultures. Congener distribution of Aroclor 1260 in the control sample (A); absolute difference in the congener distribution of Aroclor 1260 residues between the control and sediment-free culture CW-4 (B), CG-1 (C), CG-3 (D), CG-4 (E), and SG-1 (F) after 6 months incubation.

(i) In culture CW-4 (Figure 1B), PCB dechlorinators mainly attacked flanked *para*- and double flanked *meta*-chlorines from 2345-, 245-, or 234-chlorophenyl ring. In individual congener experiments, 2345-245-CB was dechlorinated mainly to 235-25-CB via 235-245-CB, and partially to 25-25-CB via 245-245-CB and 245-25-CB. 234-245-CB can be dechlorinated predominantly to 24-25-CB via 234-25-CB and 245-24-CB. The dechlorination pattern in this culture matched PCB dechlorination process H, which was first observed *in situ* both in the Acushnet Estuary (New Bedford, MA) and in parts of Hudson River (New York) [49].

(ii) In culture CG-1, dechlorinators mainly removed double flanked *meta*-chlorines from 2345- and 234-chlorophenyl rings of several most abundant congeners in Aroclor 1260 (e.g., 2345-245-CB and 234-245-CB) (Figure 1C), resulting in the accumulation of 245-245-CB, 245-24-CB. Further dechlorination of 245-245-CB to lower chlorinated PCBs was not observed after further two months of incubation. Removal of *para*-chlorine was also observed in culture CG-1 amended with 2345-245-CB, which was dechlorinated predominantly into 245-245-CB and partially into 235-245-CB. Interestingly, several *ortho*-dechlorination products were present in this culture, e.g., 236-34-CB possibly from partial dechlorination of 234-236-CB. The dechlorination pattern in culture CG-1 did not match any single existing PCB dechlorination process.

(iii) Based on appearance/disappearance of PCB congeners and their mass balance together with dechlorination of two PCB congeners (i.e., 2345-245-CB and 234-245-CB), dechlorinators in culture CG-3 mainly attacked double flanked *meta*-chlorines from 2345- and 234-chlorophenyl rings, and flanked *para*-chlorines from 2345- and 245- chlorophenyl rings (Figure 1D). For example, 2345-245-CB was dechlorinated to 235-245-CB and 25-25-CB via 245-245-CB and 245-25-CB. 234-245-CB can be dechlorinated to 24-25-CB via 245-24-CB. The dechlorination pattern in culture CG-3 could be a combination of PCB dechlorination process H (i.e., 234-, 245-, and 2345-CB) and process T (i.e., 2345-CB) (11,16).

(iv) Similar to PCB dechlorination process H in culture CW-4, dechlorinators in culture CG-4 mainly removed double flanked *meta*-chlorines from 234-chlorophenyl rings (i.e., 234-245-CB and 234-236-CB), and flanked *para*-chlorines from 2345-, and 245-chlorophenyl rings (i.e., 2345-245-CB, 2345-234-CB, 2345-25-CB and 236-245-CB) (Figure 1E). The difference between these two cultures is that culture CG-4 is unable to effectively dechlorinate 245-245-CB into lower chlorinated PCB congeners and further dechlorination of the produced penta-CBs into tetra-CBs proceeded at a slow rate.

(v) The appearance and disappearance of PCB congeners in SG-1 (Figure 1F) were quite similar with that of CW-4, both of which dechlorinated Aroclor 1260 in PCB dechlorination process H and produced 25-25-CB and 24-25-CB as predominant dechlorination products. Significant increase of 2345-26-CB was also observed in SG-1, CW-4 and CG-4, which was probably from flanked *meta*-dechlorination of 2345-236-CB. The substrate preference for chlorophenyl rings observed in these three cultures was as follows: 2345>234>245.

### Extensive dechlorination of Aroclor 1260 in sediment-free culture CG-5

**Figure 2:**
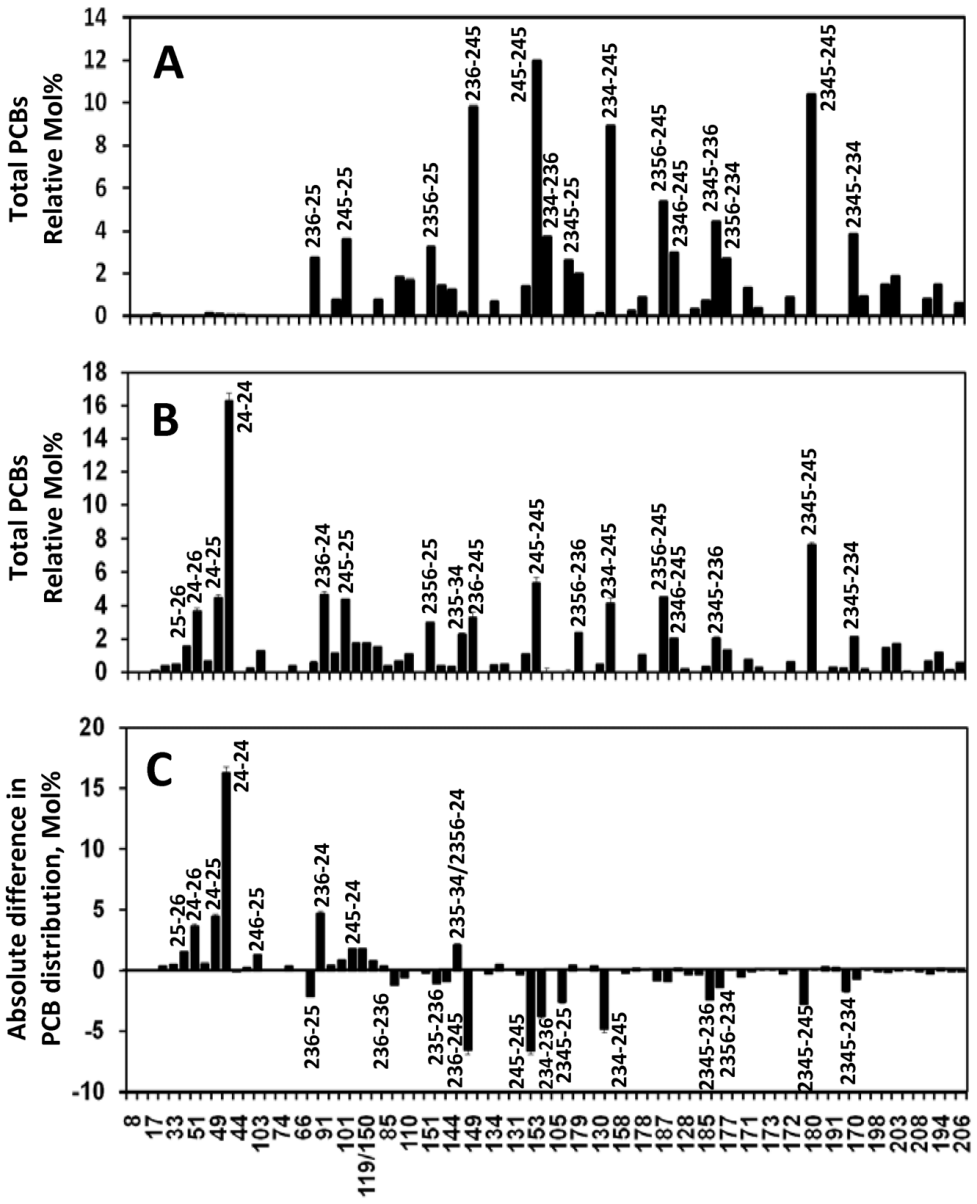
Extensive dechlorination of Aroclor 1260 in sediment-free culture CG-5. Congener distribution in the abiotic control (A) and in culture CG-5 after three months incubation (B); differences in congener distribution of Aroclor 1260 residues between the control bottles and culture CG-5 (C).

After eight serial transfers in defined medium amended with Aroclor 1260 (30 ppm) and lactate (10 mM), sediment-free culture CG-5 showed the most extensive dechlorination after three months of incubation (Figure 2) when compared with the chlorine removal of other five sediment-free cultures under the same experimental conditions. In culture CG-5, dominant hepta- (i.e., 2345-245-CB, 2345-236-CB, 2356-234-CB and 2345-234-CB) and hexa-CBs (i.e., 245-245-CB, 236-245-CB, 234-245-CB, 234-236-CB and 2345-25-CB) in Aroclor 1260 were substantially dechlorinated to lower halogenated PCB congeners, of which the prominent dechlorination products were penta- (i.e., 236-24-CB) and tetra-CBs (i.e., 24-24-CB, 24-25-CB, 24-26-CB and 25-26-CB). In contrast to microcosm CG-5 (4.46% increase of tri-CBs in Table 1), the overall tri-CBs in sediment-free CG-5 culture showed less increase (i.e., 1.11% in Table 2). Four dominant hexa-CB congeners, accounting for 71.94 mol% of total hexa-CB congeners, displayed a more than 50% decrease, i.e., 245-245-CB (55.19%), 236-245-CB (66.80%), 234-245-CB (53.37%), and 234-236-CB (100%). Four of the most abundant hepta-CB congeners also exhibited a dramatic decrease, i.e., 2345-245-CB (26.51%), 2345-236-CB (53.85%), 2345-234-CB (44.65%), and 2356-234-CB (51.12%). As shown in Table 2, the overall hepta- and hexa-CBs were significantly reduced from 36.26 mol% to 25.22 mol% (an 11.04 mol% decrease), and from 47.75 mol% to 19.52 mol% (a 28.23 mol% decrease), respectively. Accordingly, penta- and tetra-CBs as major dechlorination products increased 11.57 and 26.72 mol% of total PCBs, respectively. Culture CG-5 can dechlorinate both 2345-245-CB and 234-245-CB into 24-24-CB through attacking flanked *meta*-chlorines (i.e., 234, 245, 2345). Based on appearance/disappearance of PCB congeners and their mass balance together with dechlorination of two individual PCB congeners (i.e., 2345-245-CB and 234-245-CB), the dechlorination pattern in culture CG-5 matches PCB dechlorination process N, which occurred *in situ* in the Housatonic River [50] and was also observed in laboratory experiments with sediment slurries from Silver Lake [51], Baltimore Harbor [52], and Housatonic River [27].

**Table 2:** PCB homolog distribution in sediment-free culture CG-5 after incubating for 3 months.

PCB homolog a	Mole Percent of Total PCBs			% Decrease
	Aroclor1260 b	Dechlorinated Aroclor 1260 c	SD	
Tri-CB	0.07	1.18	0.47	
Tetra-CB	0.21	26.93	1.54	
Penta-CB	9.63	21.20	1.89	
Hexa-CB	47.75	19.52	2.10	59.12
Hepta-CB	36.26	25.22	1.33	30.44
Octa-CB	5.51	5.39	0.06	2.18
Nona-CB	0.57	0.56	0.01	

a No mono-, or dichlorobiphenyls were detected.

b Data are the means for two uninoculated controls, showing no obvious difference with original Aroclor 1260.

c Data are the means for the three CG-5 cultures that showed extensive dechlorination of Aroclor 1260.

### Identification of PCB dechlorinators

To acquire information on the possible PCB dechlorinators present in the active microcosms, PCR amplifications were conducted with primers (Table S2) specific for the 16 S rRNA genes of known PCB dechlorinating bacteria (i.e., *Dehalococcoides* and *o*-17/DF-1-type *Chloroflexi*) and the obligate dehalogenator, *Dehalobacter* species [27], [53]. To have a broader match, a new primer set (i.e., DEB165F and DEB630R in Table S2) was utilized to screen *Dehalobacter* species from these cultures, of which the specificity was verified by using RDP's ProbeMatch [54]. PCR amplification with *o*-17/DF-1 specific primers (8F/Dehal1265R in Table S2) cannot detect the involvement of *o*-17/DF-1-type *Chloroflexi* in the microcosms. All known PCB dechlorinators and the *Dehalobacter* species were absent in the two microcosms of ID-1 and MY-1 based on PCR amplification with genus-specific primers. Among the other 10 microcosms, *Dehalobacter* species were detected only in microcosm CW-1, whereas *Dehalococcoides* species were detected to be present in the other nine microcosms.

**Figure 3:**
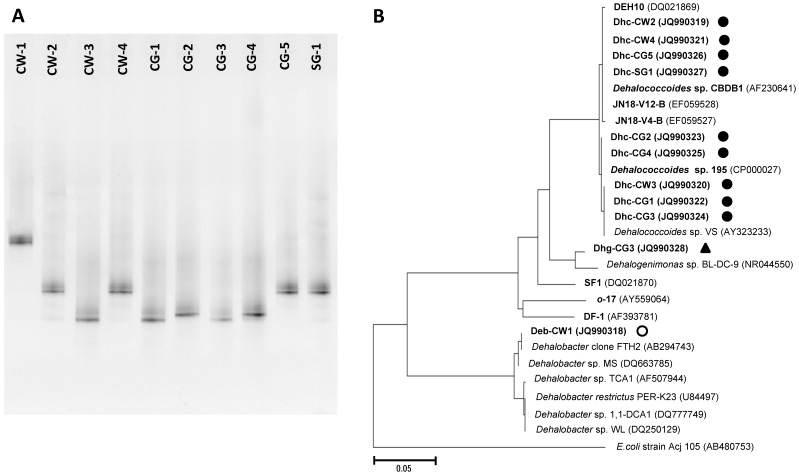
Putative PCB dechlorinating bacteria. 2S-DGGE characterization of *Dehalococcoides*/*Dehalobacter* populations present in PCB dechlorinating microcosms (A). Phylogenetic tree of PCB dechlorinating *Dehalococcoides* (closed circles), *Dehalobacter* (open circle), and *Dehalogenimonas* species (closed triangle) identified in the microcosms (B). Phylogenetic tree was calculated by neighbor-joining method using MEGA4 [46].

To phylogenetically identify these dechlorinators with nearly full-length 16 S rRNA gene sequences while avoiding tedious clone-library construction, 2S-DGGE [39] was employed to profile the *Dehalobacter* and *Dehalococcoides* populations in the microcosms (Figure 3A). The single bands present on DGGE gels suggested that *Dehalobacter*/*Dehalococcoides* bacteria in each microcosms shared identical 16 S rRNA gene sequences. The nearly full-length 16 S rRNA gene sequence (1422 bp) of *Dehalobacter* species (Deb-CW1) present in culture CW-1 shares the highest similarity of 98% with that of *Dehalobacter* clone FTH2 (AB294743) identified in a 4,5,6,7-tetrachlorophthalide dechlorinating culture [55]. As shown in Figure 3B, the identified *Dehalococcoides* bacteria were affiliated to all three *Dehalococcoides* subgroups, Cornell (cultures CG-2 and CG-4), Victoria (cultures CW-3, CG-1 and CG-3) and Pinellas (cultures CW-2, CW-4, CG-5 and SG-1). Nearly full-length 16 S rRNA gene sequences (∼1350 bp) of these *Dehalococcoides* were identical with that of representative *Dehalococcoides* (i.e., 195, VS and CBDB1) of the three subgroups except *Dehalococcoides* bacteria in culture CG1 (Dhc-CG1). The 16 S rRNA gene sequence of Dhc-CG1 shares 99% similarity (1 bp difference over 1353 bp) with that of *Dehalococcoides* sp. VS.

**Figure 4:**
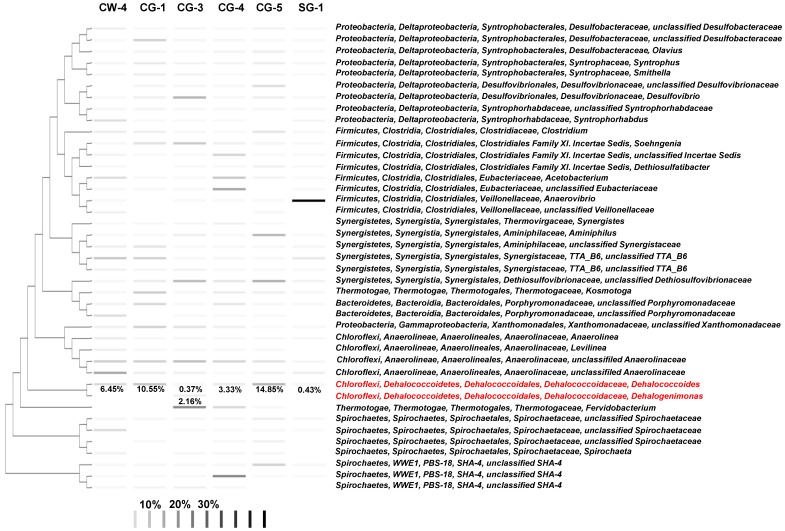
Relative abundances of predominant bacterial genera existing in 6 sediment-free PCB dechlorinating cultures. Note: the genera shown are having relative abundances higher than 1.0% in one or more of the sediment-free cultures. The band indicated the occurrence of the corresponding genus while the grayscale intensity indicated the relative abundance of the genus in the sample. Detailed relative abundance numbers were marked for the dehalogenating bacteria.

**Figure 5:**
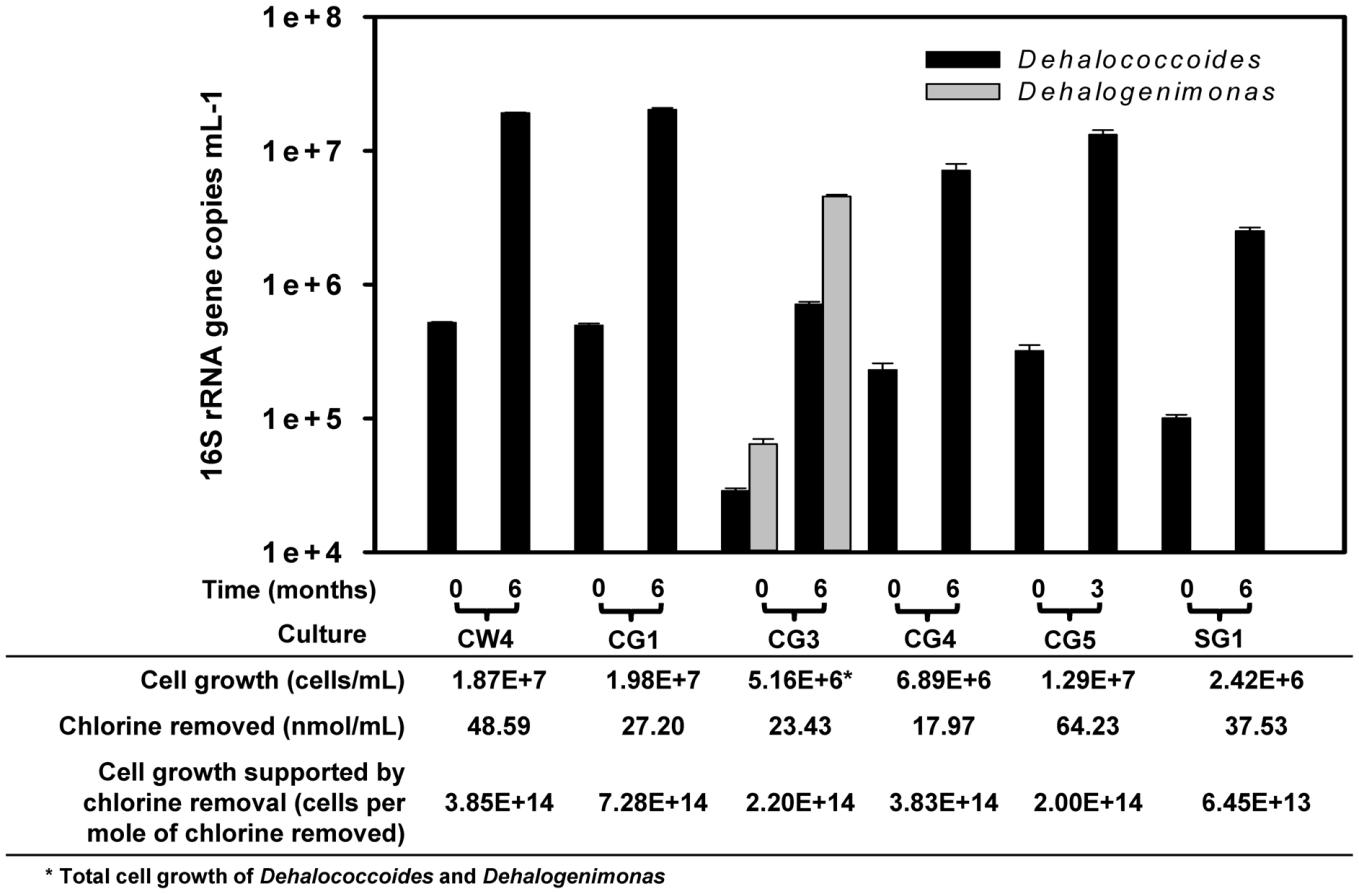
PCB-dependent growth of *Dehalococcoides*/*Dehalogenimonas* in sediment-free cultures. Cells were collected when observing their PCB dechlorination activities after 6 months of incubation for cultures CW-4, CG-1, CG-3, CG-4 and SG-1; and after 3 months for culture CG-5.

To have a comprehensive understanding of the microbial community in sediment-free PCB dechlorinating cultures, Illumina high throughput sequencing analysis was conducted to reveal their phylogenetic compositions. Results showed that the cultures were comprised mainly of the phyla Chloroflexi, Synergistetes, Firmicutes, Proteobacteria, Spirochaetes, Bacteroidetes, and Thermotogae Bacteria (Figure 4). *Dehalococcoides* within Chloroflexi phylum was the only known PCB dechlorinator present in these sediment-free cultures. Normal DGGE analysis with *Dehalococcoides*-specific GC-clamped primers (1FGC/259R in Table S2) also showed that only single 16 S rRNA genotype *Dehalococcoides* bacteria present in the sediment-free cultures (Figure S1). In particular, *Dehalococcoides* populations were present at abundances ranging from 0.37% to 14.85% of the total bacterial community, which is comparable with *Dehalococcoides* population (3.74%) in highly enriched JN culture [27]. In addition, the highest *Dehalococcoides* population percentage (14.85%) in culture CG-5 corroborated the observed fast and extensive PCB dechlorination activities. Interestingly, Illumina high throughput sequencing analysis suggested the involvement of populations from a newly characterized genus, *Dehalogenimonas* (2.16%), in reductive dechlorination of Aroclor 1260 in culture CG-3. Subsequent 2S-DGGE analysis showed the full-length 16 S rRNA gene sequence of *Dehalogenimonas* in culture CG3 (Dhg-CG3) shares 96% similarity (55 bp difference over 1493 bp) with that of *Dehalogenimonas lykanthroporepellens* BL-DC-9 (Figure 3B). qPCR analysis showed coupled growth of PCB dechlorinating bacteria (i.e. *Dehalococcoides* and *Dehalogenimonas*) with chlorine removal from PCBs in the sediment-free cultures (Figure 5). Along with an average chlorine removal of 36.49 nmol per mL (from 17.97 to 64.23 nmol per mL), the cell number of PCB dechlorinating bacteria grew to 1.1×10^7^ cells per mL (average number) in the six cultures. The resulting average growth of 3.30×10^14^ cells per mole of chlorine released is comparable to that (9.25×10^14^ cells per mole of chlorine released) in sediment-free JN cultures [28]. No obvious cell growth of PCB dechlorinators was observed in their corresponding controls that were not spiked with Aroclor 1260 (data not shown). The *Desulfovibrio* species reported to be imperative for PCB dechlorination by bacteria DF-1 [25] were also ubiquitously present in the PCB-dechlorinating sediment-free cultures (ranging from 0.01% to 13.86% of the total bacterial community).

## Discussion

In this study, sediment-free PCB-dechlorinating cultures (i.e., CW-4, CG-1, CG-3, CG-4, CG-5 and SG-1) have been successfully obtained from microcosms set up with soils, sediments and sludge, which are different from previous Aroclor1260-dechlorinating cultures requiring the presence of sediments or sediment-substitutes [8], [11], [15], [27], [29], [56]. In the six sediment-free cultures, PCB dechlorinating bacteria are able to grow on Aroclor 1260 in low PCB bioavailability without the aid of sediment-substitutes. Recently, a pure culture was also reported to debrominate octa-brominated diphenyl ether (octa-BDE) mixture in even lower bioavailability (based on concentration and water solubility of octa-BDE mixture) [57]. Therefore, some dechlorinating bacteria are able to maintain dehalogenation activities in low substrate bioavailability, which shall be easier to be isolated by using the traditional serial-dilution-to-extinction method compared to dechlorinators requiring sediment-substitutes. During our cultivation process, microcosms ID-1 and MY-1 lost their PCB dechlorination activities in the second transfer due to their low dechlorination activities, while CW-1, CW-2, CW-3 and CG-2 gradually lost their PCB dechlorination capabilities with the elimination of sediments in the serial transfers. This phenomenon may be explained by the distinct roles of sediments, e.g., supply specific nutrients for PCB dechlorinators [11], [56], and improve PCB bioavailability [8], [27]. For example, when serving to improve PCB bioavalability, sediments were replaced by silica powder to cultivate the JN culture to extensively dechlorinate Aroclor 1260 [27]. Similarly, *Dehalococcoides* sp. strain CBDB1 can also extensively dechlorinate Aroclor 1260 in the presence of silica powder when pregrown on trichlorobenzenes. However, in many PCB dechlorinating cultures, sediments likely play both roles simultaneously, since sediments can not be simply substituted with silica powder in the defined medium, e.g., PCB dechlorination activities in silica powder - dosed JN cultures can not be sustained at the third transfer unless yeast extract was added to the medium [27]. Nevertheless, during our cultivation of sediment-free cultures, appropriate carbon source selection appears to be a key factor. Comparing with acetate or/and formate used in previous studies [27]–[29], lactate serves as an excellent carbon source to support the formation of robust PCB-dechlorinating microbial consortia in the absence of sediments or sediment substitutes. This possibly is due to the lactate-supporting syntrophic bacteria (Figure 4) for maintaining PCB dechlorination by supplying metabolic elements, such as amino acids and vitamins by *Clostridium* and fatty acids by *Syntrophus*. Actually, mixed carbon sources (e.g., glucose, lactic acid, pyruvic acid and acetic acid) have been applied to defined medium in order to boost the growth of PCB dechlorinators in previous studies [11], [14]. In all, the successful cultivation of the six Aroclor 1260 - dechlorinating cultures (i.e., CW-4, CG-1, CG-3, CG-4, CG-5 and SG-1) without the presence of any sediments or sediment substitutes can facilitate future isolation and characterization of PCB dechlorinators.

Thus far, PCB dechlorination pathway has been categorized into eight different dechlorination patterns (i.e., Processes M, Q, H′, H, P, N, LP and T) [8], [10], [11], [18], [49]. In the six sediment-free cultures, three distinct PCB dechlorination patterns (e.g., H, N and T) were observed based on PCB congener profile changes from dechlorination of Aroclor 1260 and the dechlorination products of two individual PCB congeners (i.e., 2345-245-CB and 234-245-CB). Impressively, Process H is the dominant PCB dechlorination pattern observed in four of six sediment-free cultures (i.e., cultures CW-4, CG-3, CG-4 and SG-1), which implies the wide distribution of PCB dechlorination activities by Process H in the environment. Dechlorination Process H removes flanked *para*- and double flanked *meta*-chlorines (i.e., 34, 234, 245, 2345), which was first observed *in situ* both in Acushnet Estuary and in some parts of the Hudson River [45], and subsequently observed in sediment microcosms originated from Hudson River sediment [51] and in a pure culture of *Dehalococcoides* sp. CBDB1 [29]. Among the six sediment-free cultures, CG-5 showed the most extensive dechlorination of Aroclor 1260 via dechlorination Process N, showing its great potential for bioremediation applications at PCB contamination sites. For the last sediment-free culture CG-1, PCB dechlorinators mainly attack double flanked *meta*-chlorines and partially *ortho*-chlorines, which represent a novel PCB dechlorination pattern. Currently, the microbial dechlorination of *ortho*-chlorines from PCBs has not been categorized into anyone of the eight summarized PCB dechlorination processes. However, *ortho* dechlorination was observed in many cultures spiked with single PCB congeners [19], [58], [59]. A similar dechlorination pattern were also reported in anaerobic slurries of estuarine sediments amended with Aroclor 1260 [36].

Dechlorinating bacteria are normally present in PCB dechlorinating microcosms or sediment-free cultures as minor populations, e.g. *Dehalococoides* in JN cultures (3.74% of total population abundance) [28] and in sediment-free culture CG-3 (0.36% of total population abundance in this study). This poses a challenge to traditional genotyping methods (e.g., DGGE and T-RFLP) for identification of functional microbes in a mixed community [60]. Hence, this study employed Illumina high throughput sequencing together with 2S-DGGE to capture microbial community profiles and further phylogenetically characterize the key functional bacteria with full-length 16 S rRNA gene sequences. The 16 S rRNA gene-based analysis showed phylogenetically distinctive bacteria involved in reductive dechlorination of Aroclor 1260, i.e., *Dehalogenimonas* species and all three subgroups of *Dehalococcoides* (i.e., Cornell, Victoria and Pinellas). The *Dehalococcoides* bacteria are obligate dechlorinators and can only grow on halogenated compounds. Strains of the newly identified *Dehalogenimonas* genus also showed their dependent growth on halogenated compounds [61], [62]. Thus, the presence of these obligate dechlorinators suggests their involvement in PCB dechlorination. To date, knowledge about the phylogenetic information of PCB dechlorinators is still limited. All PCB dechlorinators were identified to be either *Dehalococcoides* or *o*-17/DF-1 like bacteria through culture enrichment and molecular techniques (e.g., DGGE). Furthermore, the previously identified *Dehalococcoides* bacteria for Aroclor 1260 dechlorination were all affiliated to the Pinellas subgroup, e.g., DEH10 in an Aroclor1260-dechlorinating microcosm [63], *Dehalococcoides* in JN cultures [27], [28], and *Dehalococcoides* sp. CBDB1 [29]. This study shows the involvement of all three subgroups (i.e., Cornell, Victoria and Pinellas) of *Dehalococcoides* and *Dehalogenimonas* bacteria in reductive dechlorination of Aroclor 1260. qPCR analysis further confirmed the coupled growth of *Dehalococcoides* and *Dehalogenimonas* with PCB dechlorination in sediment-free cultures. Such information would serve to broaden our knowledge of the phylogenetic diversity of PCB dechlorinators.

In conclusion, six sediment-free cultures were successfully established for reductive dechlorination of Aroclor 1260, which opened the door for following-up isolation and characterization of PCB dechlorinators. Phylogenetic analysis of the dechlorinating bacteria also expanded the diversity of known PCB dechlorinators. In the sediment-free cultures, both *Dehalococcoides* and *Dehalogenimonas* bacteria can couple their growth with PCB dechlorination. These observations would have a profound impact on cultivation and enrichment of PCB dechlorinators for future *in situ* bioremediation.

## Supporting Information

Figure S1 **DGGE profile of** ***Dehalococcoides*** **(Dhc) species present in the six sediment-free cultures with Dhc-specific GC-clamped primers 1FGC/259R.**
(TIFF)

Table S1 **Mineral salts medium compositions.**
(DOCX)

Table S2 **Primers used in this study.**
(DOCX)

## References

[1] Weber R, Gaus C, Tysklind M, Johnston P, Forter M, et al (2000) Dioxin- and POP-contaminated sites-contemporary and future relevance and challenges: overview on background, aims and scope of the series. Environ Sci Pollut Res Int 15: 363–393. 10.1007/s11356-008-0024-1 18597132

[2] Grimalt JO, van Drooge BL, Ribes A, Vilanova RM, Fernandez P, et al (2004) Persistent organochlorine compounds in soils and sediments of European high altitude mountain lakes. Chemosphere 54: 1549–1561. 14659957 10.1016/j.chemosphere.2003.09.047

[3] Xing Y, Lu Y, Dawson RW, Shi Y, Zhang H, et al (2005) A spatial temporal assessment of pollution from PCBs in China. Chemosphere 60: 731–739. 15964056 10.1016/j.chemosphere.2005.05.001

[4] Davis JA, Hetzel F, Oram JJ, McKee LJ (2007) Polychlorinated biphenyls (PCBs) in San Francisco Bay. Environ Res 105: 67–86. 17451673 10.1016/j.envres.2007.01.013

[5] Gomes HI, Dias-Ferreira C, Ribeiro AB (2013) Overview of in situ and ex situ remediation technologies for PCB-contaminated soils and sediments and obstacles for full-scale application. Sci Total Environ 445-446C: 237–260. 10.1016/j.scitotenv.2012.11.098 23334318

[6] Agency for Toxic Substances and Disease Registry (ATSDR) (2000) Toxicological profile for polychlorinated biphenyls (update).U.S. Department of Health and Human Services Agency for Toxic Substances and Disease Registry, AtlantaGA, .

[7] Chen S, Luo J, Hu M, Geng P, Zhang Y (2012) Microbial detoxification of bifenthrin by a novel yeast and its potential for contaminated soils treatment. PLoS One 7: e30862. 22348025 10.1371/journal.pone.0030862 PMC3278408

[8] Bedard DL (2008) A case study for microbial biodegradation: anaerobic bacterial reductive dechlorination of polychlorinated biphenyls-from sediment to defined medium. Annu Rev Microbiol 62: 253–270. 18729735 10.1146/annurev.micro.62.081307.162733

[9] Brown Jr JF, Wagner RE, Bedard DL, Brennan MJ, Carnahan JC, et al (1984) PCB transformations in upper Hudson sediments. Northeast Environ Sci 3: 167–169.

[10] Brown Jr JF, Bedard DL, Brennan MJ, Carnahan JC, Feng H, et al (1987) Polychlorinated biphenyl dechlorination in aquatic sediments. Science 236: 709–712. 17748310 10.1126/science.236.4802.709

[11] Wiegel J, Wu Q (2000) Microbial reductive dehalogenation of polychlorinated biphenyls. FEMS Microbiol Ecol 32: 1–15. 10779614 10.1111/j.1574-6941.2000.tb00693.x

[12] Löffler FE, Edwards EA (2006) Harnessing microbial activities for environmental cleanup. Curr Opin Biotechnol 17: 274–284. 16697178 10.1016/j.copbio.2006.05.001

[13] Quensen JF III, Tiedje JM, Boyd SA (1988) Reductive dechlorination of polychlorinated biphenyls by anaerobic microorganisms from sediments. Science 242: 752–754. 17751997 10.1126/science.242.4879.752

[14] Hartcamp-Commandeur LCM, Gerritse J, Govers HA, Parsons JR (1996) Reductive dehalogenation of polychlorinated biphenyls by anaerobic microorganisms enriched from Dutch sediments. Chemosphere 32: 1275–1286.

[15] Wu Q, Wiegel J (1997) Two anaerobic polychlorinated biphenyl-dehalogenating enrichments that exhibit different para-dechlorination specificities. Appl Environ Microbiol 63: 4826–4832. 9406402 10.1128/aem.63.12.4826-4832.1997 PMC168807

[16] Bedard DL, Van Dort H, Deweerd KA (1997) Enrichment of Microorganisms that Sequentially *meta*-, *para*-Dechlorinate the Residue of Aroclor 1260 in Housatonic River Sediment. Environ Sci Technol 31: 3308–3313.

[17] Bedard DL, Van Dort H, Deweerd KA (1998) Brominated Biphenyls Prime Extensive Microbial Reductive Dehalogenation of Aroclor 1260 in Housatonic River Sediment. Appl Environ Microbiol 64: 1786–1795. 9572952 10.1128/aem.64.5.1786-1795.1998 PMC106231

[18] Bedard DL (2003) Polychlorinated biphenyls in aquatic sediments: environmental fate and outlook for biological treatment. In:Haggblom MM, Bossert ID, Editors, Dehalogenation: Microbial Processes and Environmental Applications, Kluwer Academic Publishers, Boston, MA, pp. 443–465.

[19] Cutter L, Sowers KR, May HD (1998) Microbial dechlorination of 2,3,5,6-tetrachlorobiphenyl under anaerobic conditions in the absence of soil or sediment. Appl Environ Microbiol 64: 2966–2969. 9687458 10.1128/aem.64.8.2966-2969.1998 PMC106800

[20] Wu Q, Sowers KR, May HD (2000) Establishment of a polychlorinated biphenyl-dechlorinating microbial consortium, specific for doubly flanked chlorines, in a defined, sediment-free medium. Appl Environ Microbiol 66: 49–53. 10618202 10.1128/aem.66.1.49-53.2000 PMC91784

[21] Cutter LA, Watts JEM, Sowers KR, May HD (2001) Identification of a microorganism that links its growth to the reductive dechlorination of 2,3,5,6-chlorobiphenyl. Environ Microbiol 3: 699–709. 11846760 10.1046/j.1462-2920.2001.00246.x

[22] Wu Q, Watts JEM, Sowers KR, May HD (2002) Identification of a bacterium that specifically catalyzes the reductive dechlorination of polychlorinated biphenyls with doubly flanked chlorines. Appl Environ Microbiol 68: 807–812. 11823222 10.1128/AEM.68.2.807-812.2002 PMC126686

[23] Fennell DE, Nijenhuis I, Wilson SF, Zinder SH, Häggblom MM (2004) *Dehalococcoides ethenogenes* strain 195 reductively dechlorinates diverse chlorinated aromatic pollutants. Environ Sci Technol 38: 2075–2081. 15112809 10.1021/es034989b

[24] Beyer A, Biziuk M (2009) Environmental fate and global distribution of polychlorinated biphenyls. Rev Environ Contam Toxicol 201: 137–158. 19484591 10.1007/978-1-4419-0032-6_5

[25] May HD, Miller GS, Kjellerup BV, Sowers KR (2008) Dehalorespiration with polychlorinated biphenyls by an anaerobic ultramicrobacterium. Appl Environ Microbiol 74: 2089–2094. 18223104 10.1128/AEM.01450-07 PMC2292607

[26] Payne RB, May HD, Sowers KR (2011) Enhanced reductive dechlorination of polychlorinated biphenyl impacted sediment by bioaugmentation with a dehalorespiring bacterium. Environ Sci Technol 45: 8772–8779. 21902247 10.1021/es201553c PMC3210572

[27] Bedard DL, Bailey JJ, Reiss BL, Jerzak GV (2006) Development and characterization of stable sediment-free anaerobic bacterial enrichment cultures that dechlorinate Aroclor 1260. Appl Environ Microbiol 72: 2460–2470. 16597944 10.1128/AEM.72.4.2460-2470.2006 PMC1448987

[28] Bedard DL, Ritalahti KM, Löffler FE (2007) The *Dehalococcoides* population in sediment-free mixed cultures metabolically dechlorinates the commercial polychlorinated biphenyl mixture Aroclor 1260. Appl Environ Microbiol 73: 2513–2521. 17308182 10.1128/AEM.02909-06 PMC1855590

[29] Adrian L, Dudková V, Demnerová K, Bedard DL (2009) "*Dehalococcoides*" sp. strain CBDB1 extensively dechlorinates the commercial polychlorinated biphenyl mixture Aroclor 1260. Appl Environ Microbiol 75: 4516–4524. 19429555 10.1128/AEM.00102-09 PMC2704801

[30] Zanaroli G, Balloi A, Negroni A, Borruso L, Daffonchio D, et al (2012) A Chloroflexi bacterium dechlorinates polychlorinated biphenyls in marine sediments under in situ-like biogeochemical conditions. J Hazard Mater 209–210: 449–457. 10.1016/j.jhazmat.2012.01.042 22325634

[31] He J, Robrock KR, Alvarez-Cohen L (2006) Microbial reductive debromination of polybrominated diphenyl ethers (PBDEs). Environ Sci Technol 40: 4429–4434. 16903281 10.1021/es052508d

[32] Cole JR, Cascarelli AL, Mohn WW, Tiedje JM (1994) Isolation and characterization of a novel bacterium growing via reductive dehalogenation of 2-chlorophenol. Appl Environ Microbiol 60: 3536–3542. 7527200 10.1128/aem.60.10.3536-3542.1994 PMC201851

[33] Löffler FE, Champine JE, Ritalahti KM, Sprague SJ, Tiedje JM (1997) Complete reductive dechlorination of 1,2-dichloropropane by anaerobic bacteria. Appl Environ Microbiol 63: 2870–2875. 16535654 10.1128/aem.63.7.2870-2875.1997 PMC1389209

[34] Chu S, Hong CS (2004) Retention indexes for temperature-programmed gas chromatography of polychlorinated biphenyls. Anal Chem 76: 5486–5497. 15362911 10.1021/ac049526i

[35] Smullen LA, DeWeerd KA, Bedard DL, Fessler WA, Carnahan JC, et al. (1993) Development of a customized congener specific PCB standard for quantification of Woods Pond sediment PCBs. In *Research and Development Program for the Destruction of PCBs: twelfth progress report*.General Electric Company, Corporate Research and Development:New York,pp45–59.

[36] Wu Q, Bedard DL, Wiegel J (1997) Temperature determines the pattern of anaerobic microbial dechlorination of Aroclor 1260 primed by 2,3,4,6-tetrachlorobiphenyl in Woods Pond sediment. Appl Environ Microbiol 63: 4818–4825. 9406401 10.1128/aem.63.12.4818-4825.1997 PMC168806

[37] Chow WL (2010) Cheng D (2010) Wang S (2010) He J (2010) Identification and transcriptional analysis of *trans*-DCE-producing reductive dehalogenases in *Dehalococcoides* species. ISME J 4 1020–1030. 20357835 10.1038/ismej.2010.27

[38] He J, Ritalahti KM, Aiello MR, Löffler FE (2003) Complete detoxification of vinyl chloride by an anaerobic enrichment culture and identification of the reductively dechlorinating population as a *Dehalococcoides* species. Appl Environ Microbiol 69: 996–1003. 12571022 10.1128/AEM.69.2.996-1003.2003 PMC143667

[39] Wang S, He J (2012) Two-step denaturing gradient gel electrophoresis (2S-DGGE), a gel-based strategy to capture full-length 16 S rRNA gene sequences. Appl Microbiol Biotechnol 95: 1305–1312. 22772864 10.1007/s00253-012-4251-5

[40] Hamady M, Walker JJ, Harris JK, Gold NJ, Knight R (2008) Error-correcting barcoded primers for pyrosequencing hundreds of samples in multiplex. Nat Methods 5: 235–237. 18264105 10.1038/nmeth.1184 PMC3439997

[41] Rodrigue S, Materna AC, Timberlake SC, Blackburn MC, Malmstrom RR, et al (2010) Unlocking short read sequencing for metagenomics. PLoS One 5: e11840. 20676378 10.1371/journal.pone.0011840 PMC2911387

[42] DeSantis TZ Jr, Hugenholtz P, Keller K, Brodie EL, Larsen N, et al (2006) NAST: a multiple sequence alignment server for comparative analysis of 16 S rRNA genes. Nucleic Acids Res 34: W394–399. 16845035 10.1093/nar/gkl244 PMC1538769

[43] Hong PY, Lee BW, Aw M, Shek LP, Yap GC, et al (2010) Comparative analysis of fecal microbiota in infants with and without eczema. PLoS One 5: e9964. 20376357 10.1371/journal.pone.0009964 PMC2848600

[44] Wang Q, Garrity GM, Tiedje JM, Cole JR (2007) Naïve Bayesian Classifier for Rapid Assignment of rRNA Sequences into the New Bacterial Taxonomy. Appl Environ Microbiol 73: 5261–5267. 17586664 10.1128/AEM.00062-07 PMC1950982

[45] Altschul SF, Gish W, Miller W, Myers EW, Lipman DJ (1990) Basic local alignment search tool. J Mol Biol 215: 403–410. 2231712 10.1016/S0022-2836(05)80360-2

[46] Tamura K, Dudley J, Nei M, Kumar S (2007) MEGA4: Molecular Evolutionary Genetics Analysis (MEGA) software version 4.0. Mol Biol Evol 24: 1596–1599. 17488738 10.1093/molbev/msm092

[47] Ye L, Zhang T (2011) Pathogenic bacteria in sewage treatment plants as revealed by 454 pyrosequencing. Environ Sci Technol 45: 7173–7179. 21780772 10.1021/es201045e

[48] Yan J, Rash BA, Rainey FA, Moe WM (2009) Detection and quantification of *Dehalogenimonas* and "*Dehalococcoides*" populations via PCR-based protocols targeting 16 S rRNA genes. Appl Environ Microbiol 75: 7560–7564. 19820163 10.1128/AEM.01938-09 PMC2786429

[49] Brown Jr JF, Wagner RE (1990) PCB movement, dechlorination, and detoxication in the Acushnet Estuary. *Environ Toxicol Chem* 9: 1215–1233.

[50] Bedard DL, May RJ (1996) Characterization of the polychlorinated biphenyls in the sediments of Woods Pond: evidence for microbial dechlorination of Aroclor 1260 *in situ* . Environ Sci Technol 30: 237–245.

[51] Quensen JF III, Boyd SA, Tiedje JM (1990) Dechlorination of four commercial polychlorinated biphenyl mixtures (Aroclors) by anaerobic microorganisms from sediments. Appl Environ Microbiol 56: 2360–2369. 16348249 10.1128/aem.56.8.2360-2369.1990 PMC184734

[52] Wu Q, Sowers KR, May HD (1998) Microbial reductive dechlorination of Aroclor 1260 in anaerobic slurries of estuarine sediments. Appl Environ Microbiol 64: 1052–1058. 16349512 10.1128/aem.64.3.1052-1058.1998 PMC106366

[53] Field JA, Sierra-Alvarez R (2008) Microbial transformation and degradation of polychlorinated biphenyls. Environ Pollut 155: 1–12. 18035460 10.1016/j.envpol.2007.10.016

[54] Cole JR, Wang Q, Cardenas E, Fish J, Chai B, et al (2009) The Ribosomal Database Project: improved alignments and new tools for rRNA analysis. Nucleic Acids Res 37 (Database issue) D141–D145 doi:10.1093/nar/gkn879 19004872 10.1093/nar/gkn879 PMC2686447

[55] Yoshida N, Ye L, Baba D, Katayama A (2009) A novel *Dehalobacter* species is involved in extensive 4,5,6,7-tetrachlorophthalide dechlorination. Appl Environ Microbiol 75: 2400–2405. 19218402 10.1128/AEM.02112-08 PMC2675204

[56] Boyle AW, Blake CK, Price II WA, May HD (1993) Effects of polychlorinated biphenyl congener concentration and sediment supplementation on rates of methanogenesis and 2,3,6-trichlorobiphenyl dechlorination in an anaerobic enrichment. Appl Environ Microbiol 59: 3027–3031. 16349045 10.1128/aem.59.9.3027-3031.1993 PMC182402

[57] Ding C, Chow WL, He J (2012) Isolation of *Acetobacterium* sp. strain AG that reductively debrominates octa- and penta- brominated diphenyl ether technical mixtures. Appl Environ Microbiol doi:10.1128/AEM.02919-12 10.1128/AEM.02919-12 PMC3568622 23204415

[58] Van Dort HM, Bedard DL (1991) Reductive *ortho* and *meta* dechlorination of a polychlorinated biphenyl congener by anaerobic microorganisms. Appl Environ Microbiol 57: 1576–1578. 16348498 10.1128/aem.57.5.1576-1578.1991 PMC182990

[59] Berkaw M, Sowers KR, May HD (1996) Anaerobic *ortho* dechlorination of polychlorinated biphenyls by estuarine sedimentsfrom Baltimore Harbor. Appl Environ Microbiol 62: 2534–2539. 16535360 10.1128/aem.62.7.2534-2539.1996 PMC1388898

[60] Ding C, He J (2012) Molecular techniques in the biotechnological fight against halogenated compounds in anoxic environments. Microb Biotechnol 5: 347–367. 22070763 10.1111/j.1751-7915.2011.00313.x PMC3821678

[61] Moe WM, Yan J, Nobre MF, da Costa MS, Rainey FA (2009) *Dehalogenimonas* lykanthroporepellens gen. nov., sp. nov., a reductively dehalogenating bacterium isolated from chlorinated solvent-contaminated groundwater. Int J Syst Evol Microbiol 59: 2692–2697. 19625421 10.1099/ijs.0.011502-0

[62] Yan J, Rash BA, Rainey FA, Moe WM (2009) Isolation of novel bacteria within the Chloroflexi capable of reductive dechlorination of 1,2,3-trichloropropane. Environ Microbiol 11: 833–843. 19396942 10.1111/j.1462-2920.2008.01804.x

[63] Fagervold SK, May HD, Sowers KR (2007) Microbial reductive dechlorination of Aroclor 1260 in Baltimore harbor sediment microcosms is catalyzed by three phylotypes within the phylum Chloroflexi. Appl Environ Microbiol 73: 3009–3018. 17351091 10.1128/AEM.02958-06 PMC1892865

