# Supplementary figures and images for: Phylogenetically Distinct Bacteria Involve Extensive Dechlorination of Aroclor 1260 in Sediment-Free Cultures
Source: PLoS One. 2013 Mar 15;8(3):e59178. doi: 10.1371/journal.pone.0059178 (PMC3598663; doi:10.1371/journal.pone.0059178)

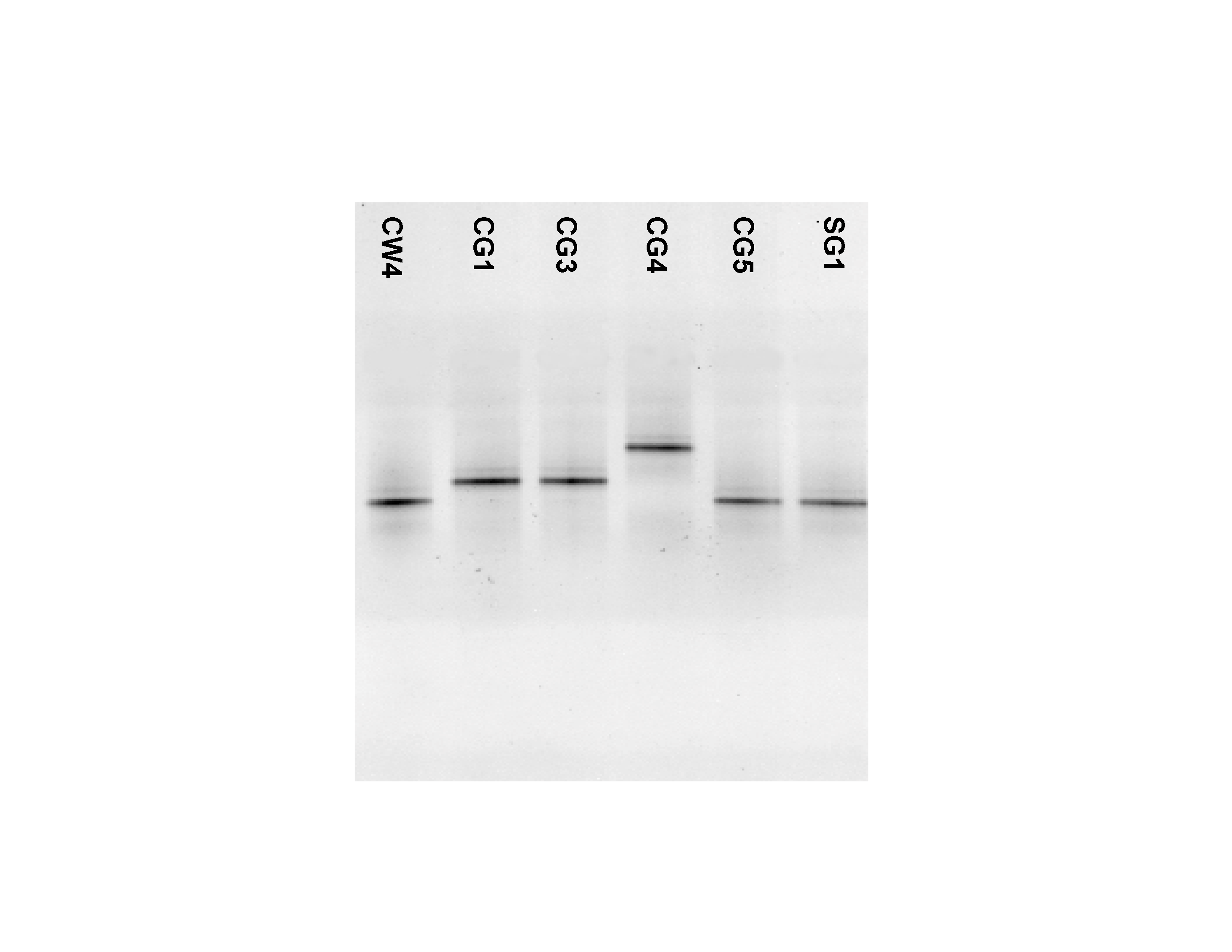

Supplement: Figure S1 — DGGE profile of Dehalococcoides (Dhc) species present in the six sediment-free cultures with Dhc-specific GC-clamped primers 1FGC/259R. (TIFF) [file pone.0059178.s001.tif]
