# Supplementary material for: Phylogenetically Distinct Bacteria Involve Extensive Dechlorination of Aroclor 1260 in Sediment-Free Cultures
Source: PLoS One. 2013 Mar 15;8(3):e59178. doi: 10.1371/journal.pone.0059178 (PMC3598663; doi:10.1371/journal.pone.0059178)
Supplement: Table S1 — Mineral salts medium compositions. (DOCX) [file pone.0059178.s002.docx]

**Table S1.** Mineral salts medium compositions.

| **Reagents** | **Amount (1L)** | |
| --- | --- | --- |
|  | ml | g |
| **Trace elements** | | |
| HCl (25% solution, w/w) | 10 | - |
| FeCl_2_·4H_2_O | - | 1.5 |
| CoCl_2_·6H_2_O | - | 0.19 |
| MnCl_2_·4H_2_O | - | 0.1 |
| ZnCl_2_ | - | 0.07 |
| H_3_BO_3_ | - | 0.006 |
| Na_2_MoO_4_·2H_2_O | - | 0.036 |
| NiCl_2_·6H_2_O | - | 0.024 |
| CuCl_2_·2H_2_O | - | 0.002 |
| **Se/W solution** | | |
| Na_2_SeO_3_·5H_2_O | - | 0.006 |
| Na_2_WO_4_·2H_2_O | - | 0.008 |
| NaOH | - | 0.5 |
| **Salt solution** | | |
| NaCl | - | 1.0 |
| MgCl_2_·6H_2_O | - | 0.5 |
| KH_2_PO_4_ | - | 0.2 |
| NH_4_Cl | - | 0.3 |
| KCl | - | 0.3 |
| CaCl_2_·2H_2_O | - | 0.015 |
| **Vitamin solutions** | | |
| Biotin | - | 0.00002 |
| Folic acid | - | 0.00002 |
| Pyridoxine hydrochloride | - | 0.0001 |
| Riboflavin | - | 0.00005 |
| Thiamine | - | 0.00005 |
| Nicotinic acid | - | 0.00005 |
| Pantothenic acid | - | 0.00005 |
| p-aminobenzoic acid | - | 0.00005 |
| Thioctic acid | - | 0.00005 |
| Vitamin B12 | - | 0.000001 |
| **Others** | | |
| TES | - | 2.292 |
| Resazurin (0.1% solution w/w) | 0.25 | - |
| DL-dithiothreitol | - | 0.0771 |
| NaHCO3 | - | 2.52 |

“-” not applicable.
