# Supplementary material for: Phylogenetically Distinct Bacteria Involve Extensive Dechlorination of Aroclor 1260 in Sediment-Free Cultures
Source: PLoS One. 2013 Mar 15;8(3):e59178. doi: 10.1371/journal.pone.0059178 (PMC3598663; doi:10.1371/journal.pone.0059178)
Supplement: Table S2 — Primers used in this study. (DOCX) [file pone.0059178.s003.docx]

**Table S2.** Primers used in this study.

| Primer | Target | Orientation | Sequence (5’ to 3’) | Annealing temperature | Reference  or source |
| --- | --- | --- | --- | --- | --- |
| 8F | *Bacteria* 16S rRNA genes | Forward | AGAGTTTGATCCTGGCTCAG | 55 [°C](http://en.wikipedia.org/wiki/Celsius)^a,b^ | 1 |
| 519F | *Bacteria* 16S rRNA genes | Forward | CAGCMGCCGCGGTAATWC | 55 [°C](http://en.wikipedia.org/wiki/Celsius) ^a,b^ | 2 |
| 518R | *Bacteria* 16S rRNA genes | Reverse | ATTACCGCGGCTGGCTGG | 55 [°C](http://en.wikipedia.org/wiki/Celsius) ^a,b^ | 3 |
| 926R | *Bacteria* 16S rRNA genes | Reverse | CCGICIATTIITTTIAGTTT | 55 [°C](http://en.wikipedia.org/wiki/Celsius) ^a,b^ | 2 |
| 1392R | *Bacteria* 16S rRNA genes | Reverse | ACGGGCGGTGTGTAC | 55 [°C](http://en.wikipedia.org/wiki/Celsius) ^a,b^ | 4 |
| DHC710R | *Dehalococcoides* 16S rRNA genes | Reverse | CAGTGTCAGTGACAACCTAG | 58 [°C](http://en.wikipedia.org/wiki/Celsius) ^a,b^ | 5 |
| DhcF | *Dehalococcoides* 16S rRNA gene | Forward | GGTAATACGTAGGAAGCAAGCG | 60 [°C](http://en.wikipedia.org/wiki/Celsius) | 6 |
| DhcR | *Dehalococcoides* 16S rRNA gene | Reverse | CCGGTTAAGCCGGGAAATT | 60 [°C](http://en.wikipedia.org/wiki/Celsius) | 6 |
| DhcProbe | *Dehalococcoides* 16S rRNA gene | Probe | VIC-ACATCCAACTTGAAAGACCACCTACGCTCACT-TAMRA | 60 [°C](http://en.wikipedia.org/wiki/Celsius) | 6 |
| 1F | *Dehalococcoides* 16S rRNA gene | Forward | A TGA ACG CTA GCG GCG | 59 [°C](http://en.wikipedia.org/wiki/Celsius) | 7 |
| 259R | *Dehalococcoides* 16S rRNA gene | Reverse | CAG ACC AGC TAC CGA TCG AA | 59 [°C](http://en.wikipedia.org/wiki/Celsius) | 7 |
| BL-DC-631f | *Dehalogenimonas* 16S rRNA gene | Forward | GGTCATCTGATACTGTTGGACTTGAGTATG | 60 [°C](http://en.wikipedia.org/wiki/Celsius) | 8 |
| BL-DC-796r | *Dehalogenimonas* 16S rRNA gene | Reverse | ACCCAGTGTTTAGGGCGTGGACTACCAGG | 60 [°C](http://en.wikipedia.org/wiki/Celsius) | 8 |
| Dehal1265R | DF-1/*o*-17 16S rRNA gene | Reverse | CCTATTGCTACCTGCTGTACC | 55 [°C](http://en.wikipedia.org/wiki/Celsius)^a,b^ | 9 |
| Dhb110f | *Dehalobacter* 16S rRNA genes | Forward | AGTAACGCGTGGGTAACCTG | 50 [°C](http://en.wikipedia.org/wiki/Celsius)^a,b^ | 10 |
| Dhb1273r | *Dehalobacter* 16S rRNA genes | Reverse | CTTCCGTCTGTACCGTCCAT | 50 [°C](http://en.wikipedia.org/wiki/Celsius)^a,b^ | 10 |
| DHCG-812R | *Dehalococcoides/Dehalogenimonas*16S rRNA genes | Reverse | GGCACAGAGAGGGTCGATACTCCC | 58 [°C](http://en.wikipedia.org/wiki/Celsius) ^a,b^ | This study |
| DEB165F | *Dehalobacter* 16S rRNA genes | Forward | CTGCTAATACCGGATGTA | 58 [°C](http://en.wikipedia.org/wiki/Celsius) ^a,b^ | This study |
| DEB630R | *Dehalobacter* 16S rRNA genes | Reverse | CGCACTTTCACATCAGACTT | 58 [°C](http://en.wikipedia.org/wiki/Celsius) ^a,b^ | 5 |
| Dhc-Cornell | *Dehalococcoides* 16S rRNA gene | Forward | GTTCATTAAAGCCGCAAGGT | 58 [°C](http://en.wikipedia.org/wiki/Celsius) ^a,b^ | 5 |
| Dhc-Victoria | *Dehalococcoides* 16S rRNA gene | Forward | TAAAGCCGTAAGGTGCTTGA | 58 [°C](http://en.wikipedia.org/wiki/Celsius) ^a,b^ | 5 |
| Dhc-Pinellas | *Dehalococcoides* 16S rRNA gene | Forward | GTTCACTAAAGCCGTAAGGC | 58 [°C](http://en.wikipedia.org/wiki/Celsius) ^a,b^ | 5 |
| GC-clamp^c^ | - | Forward | CGCCCGCCGCGCGCGGCGGGCGGGGCGGGGGCACGGGGGG | - ^a,d^ | 3 |

^a^ Normal PCR reaction conditions: the PCR solution (100 μl) mixture contained 1× buffer solution, 2.5 mM of MgCl_2_, 0.13mg/ml of BSA, 0.25 mM of each dNTPs, 50 nM of each primer, 1 U of Taq DNA polymerase (QIAGEN, GmbH, Germany).

^b^ The thermal program included initial denaturation at 95°C for 5 min; followed by 30 cycles of 1.0 min at 95°C, 1.0 min annealing at abovementioned temperature, and 1.0 min at 72°C; and a final extension step of 5 min at 72°C.

^c^ The GC-clamp was attached to the 5´-end of the 1F, 8F and 519F to get primers 1FGC, 8FGC and 519FGC, respectively.

^d^ The PCR amplification was carried out with a touchdown thermocycling program: initial denaturation (95[°C](http://en.wikipedia.org/wiki/Celsius), 5 min); 20 cycles of 95[°C](http://en.wikipedia.org/wiki/Celsius) (1 min), annealing (decreasing from 65[°C](http://en.wikipedia.org/wiki/Celsius) to 55[°C](http://en.wikipedia.org/wiki/Celsius) at -0.5[°C](http://en.wikipedia.org/wiki/Celsius)/cycle, 1 min), and 72[°C](http://en.wikipedia.org/wiki/Celsius) for 1 min 30 s; an additional 20 cycles with annealing at 55[°C](http://en.wikipedia.org/wiki/Celsius); and final extension (72[°C](http://en.wikipedia.org/wiki/Celsius), 5 min).

1. Reysenbach AL, Wickham GS, Pace NR (1994) Phylogenetic analysis of the hyperthermophilic pink filament community in Octopus Spring, Yellowstone National Park. Appl Environ Microbiol 60: 2113-2119.

2. Baker GC, Smith JJ, Cowan DA (2003) Review and re-analysis of domain-specific 16S primers. J Microbiol Methods 55: 541-555.

3. Muyzer G, De Waal EC, Uitterlinden AG (1993) Profiling of complex microbial populations by denaturing gradient gel electrophoresis analysis of polymerase chain reaction-amplified genes coding for 16S rRNA. Appl Environ Microbiol 59: 695-700.

4. Lane DJ (1991) 16S/23S rRNA sequencing. In *Nucleic acid techniques in bacterial systematics*; Stackebrandt, E., Goodfellow, M., Eds.; John Wiley & Sons, Chichester, pp 115-175.

5. Wang S, He J (2012) Two-step denaturing gradient gel electrophoresis (2S-DGGE), a gel-based strategy to capture full-length 16S rRNA gene sequences. Appl Microbiol Biotechnol 95: 1305-1312.

6. Holmes VF, He J, Lee PK, Alvarez-Cohen L (2006) Discrimination of multiple *Dehalococcoides* strains in a trichloroethene enrichment by quantification of their reductive dehalogenase genes. Appl Environ Microbiol 72: 5877-5883.

7. Duhamel M, Mo K, Edwards EA (2012) Characterization of a highly enriched dehalococcoides-containing culture that grows on vinyl chloride and trichloroethene. Appl Environ Microbiol 70: 5538-5545.

8. Yan J, Rash BA, Rainey FA, Moe WM (2009b) Detection and quantification of *Dehalogenimonas* and "*Dehalococcoides*" populations via PCR-based protocols targeting 16S rRNA genes. Appl Environ Microbiol 75: 7560-7564.

9. Watts JE, Fagervold SK, May HD, Sowers KR (2005) A PCR-based specific assay reveals a population of bacteria within the Chloroflexi associated with the reductive dehalogenation of polychlorinated biphenyls. Microbiology 151: 2039-2046.

# 10. [Nelson JL](http://www-ncbi-nlm-nih-gov.libproxy1.nus.edu.sg/pubmed?term=Nelson%20JL%5BAuthor%5D&cauthor=true&cauthor_uid=21732639), [Fung JM](http://www-ncbi-nlm-nih-gov.libproxy1.nus.edu.sg/pubmed?term=Fung%20JM%5BAuthor%5D&cauthor=true&cauthor_uid=21732639), [Cadillo-Quiroz H](http://www-ncbi-nlm-nih-gov.libproxy1.nus.edu.sg/pubmed?term=Cadillo-Quiroz%20H%5BAuthor%5D&cauthor=true&cauthor_uid=21732639), [Cheng X](http://www-ncbi-nlm-nih-gov.libproxy1.nus.edu.sg/pubmed?term=Cheng%20X%5BAuthor%5D&cauthor=true&cauthor_uid=21732639), [Zinder SH](http://www-ncbi-nlm-nih-gov.libproxy1.nus.edu.sg/pubmed?term=Zinder%20SH%5BAuthor%5D&cauthor=true&cauthor_uid=21732639) (2011) A role for Dehalobacter spp. in the reductive dehalogenation of dichlorobenzenes  and monochlorobenzene. [Environ Sci Technol](http://www-ncbi-nlm-nih-gov.libproxy1.nus.edu.sg/pubmed?term=A%20Role%20for%20Dehalobacter%20spp.%20in%20the%20Reductive%20Dehalogenation%20of%20Dichlorobenzenes%20and%20Monochlorobenzene) 45:6806-6813.
